# Epidemiological Insights Into BVDV and IBRV in Feeder Cattle After Cross‐Regional Transport in China 2022–2024: Coinfection Dynamics and Implications for BRDC Control

**DOI:** 10.1155/tbed/3614383

**Published:** 2026-04-13

**Authors:** Li Ren, Changxiao Tian, Cheng Shen, Yanxia Hao, Xijun Yan, Zhicai Zuo

**Affiliations:** ^1^ Department of Clinical Veterinary, Key Laboratory of Animal Disease and Human Health of Sichuan Province, College of Veterinary Medicine, Sichuan Agricultural University, Chengdu, Sichuan, 611130, China, sicau.edu.cn; ^2^ Research and Development Department, Sinovet (Jiangsu) Biopharmaceuticals Co. Ltd, Taizhou, Jiangsu, 225300, China

**Keywords:** bovine viral diarrhea virus, BRDC, cross-regional transport, epidemiology, infectious bovine rhinotracheitis virus

## Abstract

**Background:**

Bovine viral diarrhea virus (BVDV) and infectious bovine rhinotracheitis virus (IBRV) are key components of the bovine respiratory disease complex (BRDC) in China’s cross‐regional feeder system. We quantified antibody and antigen positivity and identified epidemiological determinants across provinces, seasons, years, and clinical status.

**Methods:**

We collected 7592 sera and 6651 clinical specimens from five provinces spanning pretransport (north) and posttransport (southwest) settings across all seasons from 2022 to 2024. Seroprevalence and antigen prevalence were summarized by province, year, season, and clinical status. Logistic regression estimated adjusted odds ratios (aORs) for single‐virus outcomes; multinomial models (adjusted relative risk ratios [aRRRs]) and modified Poisson models (adjusted prevalence ratios [aPRs]) evaluated antibody coinfection.

**Results:**

Overall, 78.85% of sera were BVDV‐antibody positive (95% CI 77.99–79.69) and 70.11% were IBRV‐antibody positive (95% CI 69.07–71.14). Seroprevalence was higher in the southwest (Sichuan/Chongqing) than in the north (Hebei/Inner Mongolia/Liaoning), rose from 2022 to 2024, and peaked in winter; cattle without clinical signs showed higher seroprevalence than symptomatic animals. Antigen positivity was lower overall—BVDV 7.01% (95% CI 6.40–7.65) and IBRV 5.79% (95% CI 5.24–6.38)—and was greater in the southwest and in winter. In multivariable models, BVDV seropositivity was strongly associated with residence in Sichuan (aOR 2.37, 95% CI 1.89–2.99) and Chongqing (aOR 2.86, 95% CI 2.10–3.91) versus Hebei, and with winter (aOR 1.64, 95% CI 1.38–1.96) versus spring; clinical signs were inversely associated (aOR 0.83, 95% CI 0.73–0.95). For antigens, IBRV positivity increased in 2024 vs. 2022 (aOR 1.53, 95% CI 1.16–2.02) and was higher in clinically symptomatic cattle (aOR 1.96, 95% CI 1.54–2.50). Coinfection analyses showed the greatest departure from the double‐negative reference for the BVDV−/IBRV+ category (aRRR 0.500, 95% CI 0.318–0.785; *p*  < 0.01).

**Conclusions:**

Under China’s cross‐regional finishing model, BVDV/IBRV circulate widely, with higher risk in southwestern provinces, in winter, and in recent years. Antigen detection concentrates among symptomatic cattle, underscoring the value of paired serology—PCR at intake and calendar‐aware vaccination and management before winter moves. These findings support risk‐based BRDC prevention aligned to province and season.

## 1. Introduction

Bovine respiratory disease complex (BRDC) remains a major health issue in intensively managed beef systems, driving mortality, antimicrobial use, and substantial economic losses [1–6]. The etiology of BRDC involves complex interactions between host, pathogen, environment, and management factors. While many pathogens, including viruses and bacteria, have been implicated in BRDC, the most common viral pathogens associated with BRDC include bovine viral diarrhea virus (BVDV), infectious bovine rhinotracheitis virus (IBRV), and bovine respiratory syncytial virus (BRSV) [7–10]. BVDV causes transient infection, reproductive losses, and—critically—the creation of persistently infected (PI) animals that shed continuously and can move undetected through markets unless specifically tested [11]. IBRV establishes lifelong latency with stress‐related reactivation, enabling silent spread during transport, at markets, and at receiving hubs [9, 12]. Its role in shipping fever is well recognized, and coinfection with BVDV can amplify clinical severity and transmission potential [13–16].

The risk of BRDC escalates when cattle are assembled from multiple sources and moved over long distances. This configuration now typifies China’s cross‐regional finishing chain: animals are procured in northern production areas and shipped to large receiving yards in the southwest [17]. In practice, the northern supply system involves multichannel—young cattle are bought from numerous smallholder farmers, aggregated and commingled at livestock trading markets, and only then consigned southward. Upon arrival, pens often contain cattle of mixed and partly unknown origin, with incomplete movement histories, uncertain vaccination records, and limited capacity for lot‐level traceback. In conclusion, transport stress, crowding at markets, and rapid remixing at arrival create favorable conditions for transmission and clinical disease.

Current evidence from China predominantly stems from farm‐level or outbreak investigations, with limited adjustment for movement context, market commingling, clinical status at intake, or coinfection structures [18, 19]. As the cross‐regional finishing model expands, decision‐makers need estimates that link place (origin provinces vs. southwestern hubs) and time (year, season) with both serologic and antigenic markers under real‐world conditions of multisource procurement and imperfect records. Even with vaccination, heterogeneous coverage, antigenic diversity, and mixing of unverified lots along the marketing chain continue to facilitate viral introductions [20, 21]. Serology reflects prior exposure or vaccine‐derived immunity, whereas antigen/PCR indicates ongoing infection; interpreted together, they offer a workable picture of population risk in a mobile, commingled system [22]. Despite extensive global literature, contemporary system‐level estimates for BVDV in China’s finishing network—stratified by province, calendar year, and season—remain sparse.

To address these research gaps, we conducted a multiprovincial survey spanning five key provinces that anchor the north‐to‐south movement chain. Our objectives were to (i) quantify seroprevalence and antigen positivity rates for BVDV and IBRV in cattle upon entry to or present within finishing operations; (ii) estimate adjusted associations with province, year, season, and clinical status using logistic models; and (iii) characterize serologic and antigen coinfection using multinomial and modified Poisson approaches. By pairing serology with antigen testing in a setting of market aggregation, mixed origin, and uncertain vaccination background, we aim to provide evidence to support PI detection at origin, risk‐stratified vaccination, and calendar‐aware intake biosecurity in China’s finishing sector.

## 2. Materials and Methods

### 2.1. Study Setting and Design

We conducted a multiregional, cross‐sectional survey of feeder cattle between 2022 and 2024 in five Chinese provinces representing northern source regions (Hebei, Inner Mongolia, and Liaoning) and southwestern finishing hubs (Sichuan and Chongqing). In total, 7592 sera and 6651 clinical specimens were collected. Northern samples were obtained at large trading markets; southwestern samples were taken at provincial/municipal transit stations and at farms receiving cattle from the north. Northern provinces included in this study serve primarily as source regions for feeder cattle, where animals are procured from multiple smallholder farms and aggregated at livestock markets prior to long‐distance transportation. As a result, larger numbers of animals are available and accessible for sampling at these aggregation points. In contrast, southwestern provinces represent receiving and finishing regions, where cattle arrive in batches and are distributed across commercial farms with varying herd sizes, leading to comparatively smaller sample sizes.

### 2.2. Sampling and Data Collection

**Figure 1:**
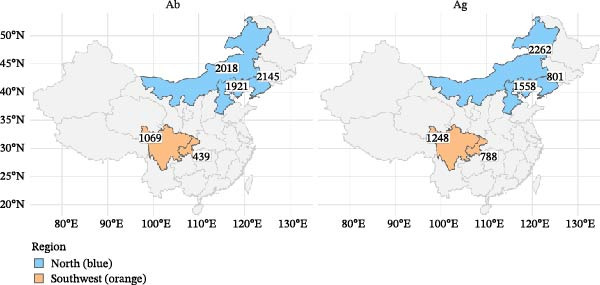
Sampling counts by province. Blue represents the north, and orange represents the south.

Sampled animals were Simmental bulls awaiting sale, weighing ~250 kg. Sampling followed local routine management and transport schedules. For analyses, observations were stratified by province, year (2022–2024), season (spring, summer, autumn, and winter), and clinical status at sampling (presence/absence of clinical signs—nasal discharge and coughing). Region (north vs. southwest) was used descriptively. No repeated sampling of the same animal was conducted in this study. Each animal was sampled only once at the time of enrollment. Detailed province sample information is provided in Table 1 and Figure 1.

**Table 1:** Antibody and antigen samples collection information in different provinces from 2022 to 2024.

Provinces	Antibody samples			Antigen samples		
	2022	2023	2024	2022	2023	2024
Hebei	492	515	914	385	447	720
Liaoning	511	751	883	439	161	201
Inner Mongolia	414	602	1002	603	819	840
Sichuan	262	377	430	496	515	237
Chongqing	134	225	80	141	324	323
Total	1813	2470	3309	2064	2266	2321

### 2.3. Laboratory Testing

Antibodies to BVDV and IBRV were detected using ELISA kits (BVDV p80 competitive ELISA; IBRV indirect ELISA; Aidivir, Qingdao) according to the manufacturers’ instructions. Antigen detection for both viruses used real‐time PCR kits (Era Biology, Beijing). All assays followed kit cutoffs and quality controls. For BVDV, amplification targeted the 5^′^ untranslated region (5^′^UTR) of the viral genome. For IBRV, amplification targeted the glycoprotein B (gB) gene. Each reaction was carried out in a total volume of 20 μL, consisting of PCR reaction mix, primers, probes, enzyme, and 2–5 μL of extracted nucleic acid, according to the manufacturer’s instructions. The thermal cycling conditions were as follows: an initial denaturation at 95°C for 30 s, followed by 40 cycles of denaturation at 95°C for 5 s and annealing/extension at 60°C for 30 s, with fluorescence signal acquisition at the end of each cycle.

### 2.4. Outcomes and Definitions

Binary outcomes were seropositivity (BVDV or IBRV) and antigen positivity (BVDV or IBRV). For coinfection, we defined four mutually exclusive categories using BVDV−/IBRV− as the reference: BVDV+/IBRV−, BVDV−/IBRV+, and BVDV+/IBRV+.

### 2.5. Data Processing and Analysis

The seropositivity and antigen positivity rates of BVDV and IBRV were calculated. Chi‐square tests and multivariable logistic regression analyses were performed using SPSS software to assess differences in detection rates across different regions, seasons, clinical status, and years, as well as to analyze the coinfection patterns of the two pathogens.

### 2.6. Exposures and Covariates

Prespecified covariates included province (reference = Hebei), year (reference = 2022), season (reference = spring), and clinical status (reference = no clinical signs). These covariates were chosen a priori on biological and management grounds.

### 2.7. Statistical Analysis

We summarized proportions with 95% confidence intervals and compared strata using chi‐square tests where appropriate. Multivariable logistic regression estimated adjusted odds ratios (aORs) for seropositivity and antigen positivity, adjusting for province, year, season, and clinical status. For the four‐level coinfection outcome, multinomial logistic regression estimated adjusted relative risk ratios (aRRRs) relative to double‐negative cattle. Given the high prevalence of some outcomes, modified Poisson regression with robust standard errors was used as a sensitivity analysis to obtain adjusted prevalence ratios (aPRs). We examined multicollinearity (e.g., VIF) and overall model fit, and report effect sizes with 95% CI; *p* values are two‐sided, given to three decimals (*p*  < 0.001 when applicable). Core modeling was performed in SPSS; figures and maps were produced in R (4.5.1).

### 2.8. Mapping and Figure Preparation

Provincial maps were drawn from publicly available administrative boundaries. Figure panels use consistent axes and color schemes; legends specify that points denote effect estimates, bars denote 95% CI, and the dashed vertical line marks 1.00 for odds/ratio plots.

## 3. Result

### 3.1. Study Population

In total, 7592 sera and 6651 clinical specimens were collected across five provinces (2022–2024), covering pretransport (north) and posttransport (southwest) settings over all seasons. This structure supports province‐, year‐, and season‐specific estimates and underpins the multivariable models presented below (Table 2).

**Table 2:** Distribution of BVDV and IBRV antibody and antigen testing samples by clinical status, season, region, and year.

Clinical status	Distribution		Antibody % (*n*/*N*)	Antigen % (*n*/*N*)
	Basis for classification	Detailed classification		
No	Season	Spring	22.18 (770/3471)	23.9 (668/2795)
		Summer	13.97 (485/3471)	10.8 (301/2795)
		Autumn	33.13 (1150/3471)	35.4 (990/2795)
		Winter	30.71 (1066/3471)	29.9 (836/2795)
	Region	North	68.95 (2428/3471)	51.7 (1445/2795)
		South	30.05 (1043/3471)	48.3 (1350/2795)
	Year	2022	38.40 (1333/3471)	30 (838/2795)
		2023	17.55 (609/3471)	34.6 (967/2795)
		2024	44.05 (1529/3471)	35.4 (990/2795)
Yes	Season	Spring	24.63 (1015/4121)	14.2 (548/3856)
		Summer	13.22 (545/4121)	15.8 (608/3856)
		Autumn	37.08 (1528/4121)	21.8 (840/3856)
		Winter	25.07 (1033/4121)	48.2 (1860/3856)
	Region	North	88.72 (3656/4121)	82.2 (3170/3856)
		South	11.28 (465/4121)	17.8 (686/3856)
	Year	2022	11.65 (480/4121)	31.8 (1226/3856)
		2023	45.16 (1861/4121)	33.7 (1299/3856)
		2024	43.19 (1780/4121)	34.5 (1331/3856)

### 3.2. Antibody Seroprevalence and Adjusted Associations BVDV and IBRV

Among 7592 sera, 78.85% were BVDV‐antibody positive and 70.11% were IBRV‐antibody positive (Table 3). Seroprevalence was consistently higher in the southwest (Sichuan and Chongqing) than in the north (Hebei, Inner Mongolia, and Liaoning), increased from 2022 to 2024, and peaked in winter. Cattle without clinical signs showed higher seroprevalence than symptomatic animals. Stratified estimates are provided in Table 3 and Figure 2.

Figure 2Seroprevalence of BVDV and IBRV antibodies by province, year, season, and clinical status. (A) Spatial distribution of BVDV and IBRV antibody positivity in cattle across Chinese provinces. (B) Comparison of BVDV and IBRV antibody positivity by epidemiological factors. Green represents—BDBV; orange represents IBRV. (C) BVDV Ab positivity (%). (D) IBRV Ab positivity (%). (A–D) BVDV and IBRV; bars/points depict proportions with 95% CI. Overall seroprevalence: BVDV 78.85% (95% CI 77.99–79.69); IBRV 70.11% (95% CI 69.07–71.14).

**Figure:**
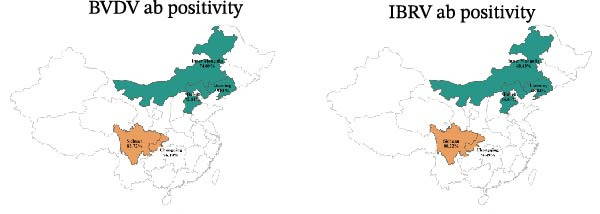
(A)

**Figure:**
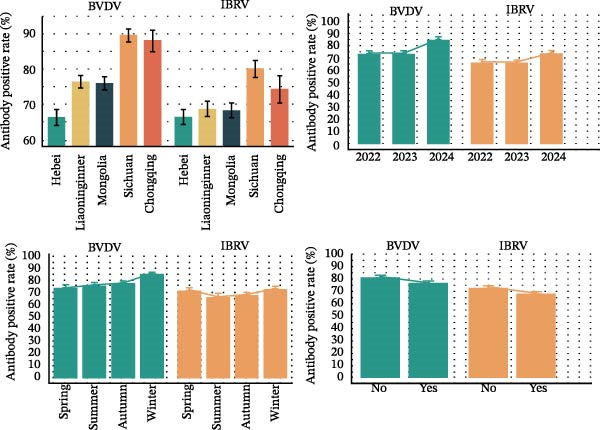
(B)

**Figure:**
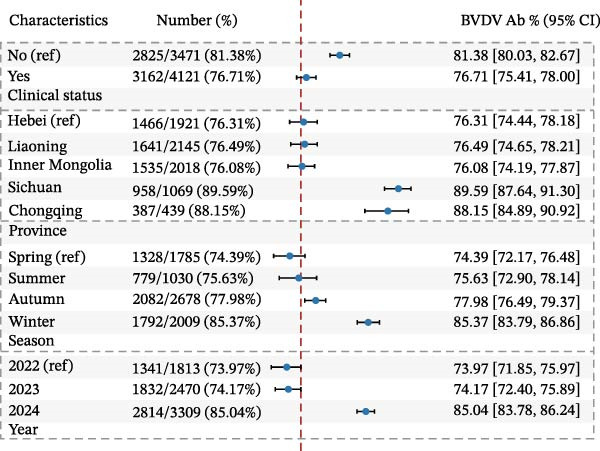
(C)

**Figure:**
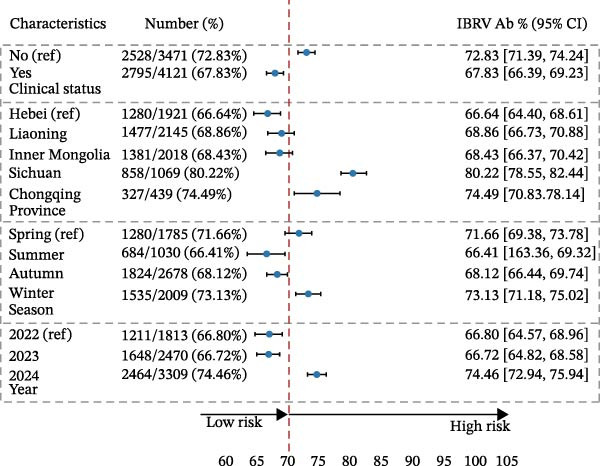
(D)

**Table 3:** Seroprevalence of BVDV and IBRV antibodies by province, year, season, and clinical status.

Distribution	Item	BVDV Ab % (95% CI)	IBRV Ab % (95% CI)
Overall	All	78.85 (77.99–79.69)	70.11 (69.07–71.14)
Province	Hebei	76.31 (74.44–78.18)	66.64 (64.40–68.61)
	Liaoning	76.49 (74.65–78.21)	68.86 (66.73–70.88)
	Inner Mongolia	76.08 (74.19–77.87)	68.43 (66.37–70.42)
	Sichuan	89.59 (87.64–91.30)	80.22 (78.55–82.44)
	Chongqing	88.15 (84.89–90.92)	74.49 (70.83–78.14)
Year	2022	73.97 (71.85–75.97)	66.80 (64.57–68.96)
	2023	74.17 (72.40–75.89)	66.72 (64.82–68.58)
	2024	85.04 (83.78–86.24)	74.46 (72.94–75.94)
Season	Spring	74.39 (72.17–76.48)	71.66 (69.38–73.78)
	Summer	75.63 (72.90–78.14)	66.41 (63.36–69.32)
	Autumn	77.98 (76.49–79.37)	68.12 (66.44–69.74)
	Winter	85.37 (83.79–86.86)	73.13 (71.18–75.02)
Clinical status	No	81.38 (80.03–82.67)	72.83 (71.39–74.24)
	Yes	76.71 (75.41–78.00)	67.83 (66.39–69.23)

*Note:* Values are % with 95% confidence intervals; *n* per stratum provided.

The univariable and multivariable logistic regression analyses identified significant spatial, temporal, and clinical determinants of BVDV and IBRV antibody seropositivity (Table 4).

**Table 4:** Results of univariable and multivariable logistic regression analyses of potential risk factors associated with BVDV and IBRV seropositivity.

Factor	Level	OR (95% CI)	*p*	aOR (95% CI)	*p*	OR (95% CI)	*p*	aOR (95% CI)	*p*
		BVDV				IBRV			
Province	Hebei (ref)	1.0	—	1.0	—	1.0	—	1.0	—
	Liaoning	1.01 (0.87–1.17)	0.89	1.11 (0.95–1.29)	0.207	1.11 (0.97–1.26)	0.13	1.24 (1.08–1.43)	0.002
	Inner Mongolia	0.99 (0.85–1.14)	0.86	0.98 (0.84–1.14)	0.770	1.09 (0.95–1.24)	0.23	1.09 (0.95–1.25)	0.23
	Sichuan	2.68 (2.14–3.35)	<0.001	2.37 (1.89–2.99)	<0.001	2.04 (1.70–2.43)	<0.001	2.09 (1.74–2.52)	<0.001
	Chongqing	2.31 (1.69–3.14)	<0.001	2.86 (2.10–3.91)	<0.001	1.46 (1.16–1.85)	0.002	1.63 (1.28–2.07)	<0.001
Year	2022 (ref)	1.0	—	1.0	—	1.0	—	1.0	—
	2023	1.01 (0.88–1.16)	0.88	1.09 (0.93–1.27)	0.306	1.00 (0.88–1.13)	0.96	1.10 (0.96–1.27)	0.18
	2024	2.00 (1.74–2.31)	<0.001	2.26 (1.93–2.63)	<0.001	1.45 (1.28–1.64)	<0.001	1.60 (1.40–1.83)	<0.001
Season	Spring (ref)	1.0	—	1.0	—	1.0	—	1.0	—
	Summer	1.07 (0.89–1.28)	0.47	1.19 (0.99–1.43)	0.073	0.94 (0.85–1.04)	0.24	0.78 (0.66–0.92)	0.005
	Autumn	1.22 (1.06–1.44)	0.006	1.09 (0.91–1.30)	0.318	0.84 (0.73–0.96)	0.012	0.75 (0.62–0.90)	0.002
	Winter	2.01 (1.71–2.36)	<0.001	1.64 (1.38–1.96)	<0.001	1.46 (1.29–1.65)	<0.001	1.19 (1.04–1.40)	0.014
Clinical status	No (ref)	1.0	—	1.0	—	1.0	—	1.0	—
	Yes	0.75 (0.67–0.84)	<0.001	0.83 (0.73–0.95)	0.007	0.79 (0.71–0.87)	<0.001	0.82 (0.73–0.92)	<0.001

*Note:* Models adjust for province, year, season, and clinical status; aOR with 95% CI and *p* values are shown.

In multivariable logistic models (adjusted for province, year, season, and clinical status), BVDV seropositivity was higher in Sichuan (aOR 2.37, 95% CI 1.89–2.99) and Chongqing (aOR 2.86, 95% CI 2.10–3.91) compared with Hebei. Sampling in 2024 (vs. 2022) and in winter was associated with increased odds (e.g., aOR 2.26, 95% CI 1.93–2.63 for 2024; aOR 1.64, 95% CI 1.38–1.96 for winter). Cattle with clinical signs had lower odds of BVDV seropositivity (aOR 0.83, 95% CI 0.73–0.95). For IBRV, provinces Liaoning (aOR 0.65, 95% CI 0.44–0.96) and Inner Mongolia (aOR 0.56, 95% CI 0.41–0.73) had lower odds relative to Hebei. Odds were higher in 2024 than 2022 (aOR 1.60, 95% CI 1.40–1.83). After adjustment, seasonal terms were not significant. As with BVDV, cattle with clinical signs showed lower odds of IBRV seropositivity (aOR 0.82, 95% CI 0.73–0.92). Corresponding forest plots are provided in Figure 3.

Figure 3Multivariable logistic regression analyses of potential risk factors associated with BVDV and IBRV seropositivity. (A) Forest plot of adjusted prevalence odds ratios (aORs) for BVDV antibody positivity by epidemiological factors. (B) Forest plot of adjusted prevalence odds ratios (aORs) for IBRV antibody positivity by epidemiological factors. Forest plots display aORs with 95% CI; dashed vertical line at 1.00. Examples (BVDV): Sichuan vs. Hebei aOR 2.37 (1.89–2.99); Chongqing vs. Hebei aOR 2.86 (2.10–3.91); winter vs. spring aOR 1.64 (1.38–1.96); clinical signs (Yes) aOR 0.83 (0.73–0.95).

**Figure:**
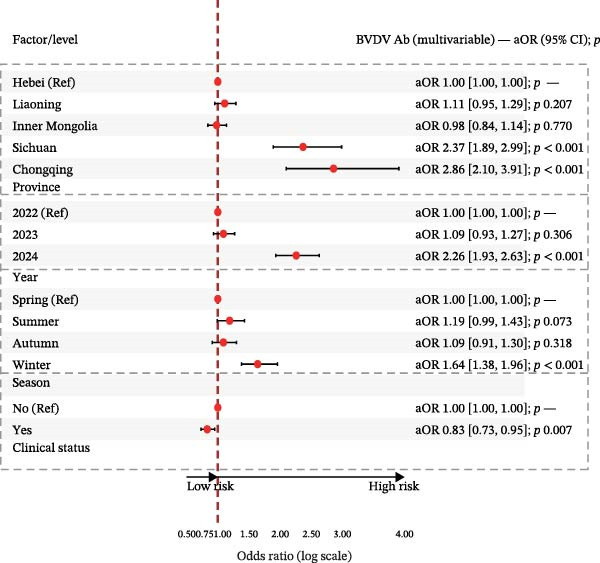
(A)

**Figure:**
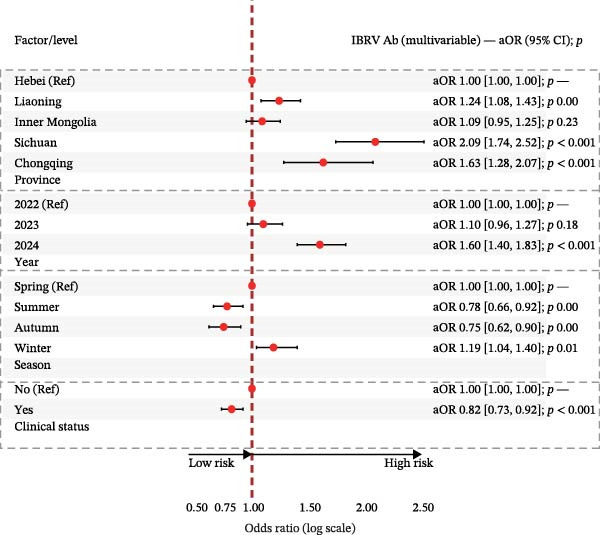
(B)

### 3.3. Serologic Coinfection (Multinomial Model)

In multinomial models with BVDV−/IBRV− as the reference and adjustment for province, year, season, and clinical status, all nonreference categories showed higher relative risk (Table 5; Figure 4).

Figure 4Adjusted relative risk ratios (aRRR) and adjusted prevalence ratios (aPR) for antibody co‐infection. (A) Forest plot of adjusted relative risk ratio (aRRR) for BVDV antigen positivity by epidemiological factors. (B) Forest plot of adjusted relative risk ratios (aPR) for IBRV antigen positivity by epidemiological factors. Outcome categories: BVDV−/IBRV− (reference), BVDV+/IBRV−, BVDV−/IBRV+, BVDV+/IBRV+. Points = aRRR (or aPR where shown); bars = 95% CI; dashed vertical line at 1.00. Largest effect observed for BVDV−/IBRV+ (aRRR 1.94, 95% CI 1.49–2.53; *p* < 0.001).

**Figure:**
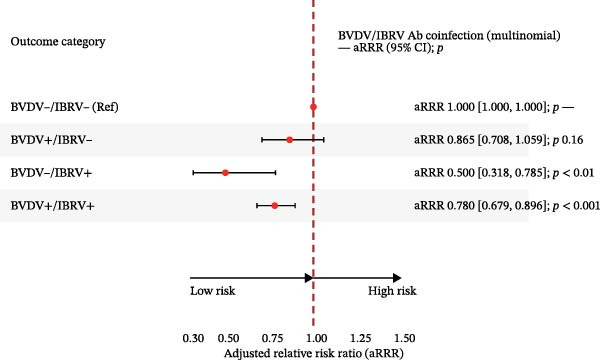
(A)

**Figure:**
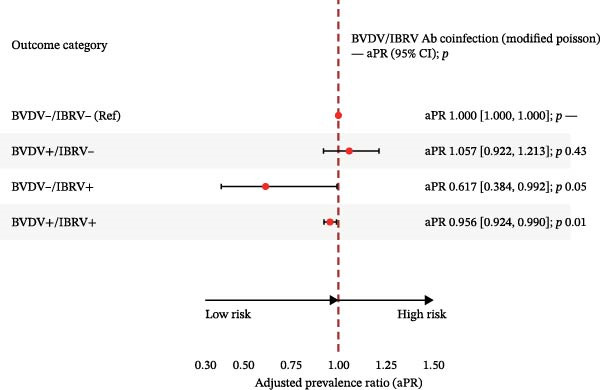
(B)

**Table 5:** Adjusted relative risk ratios (aRRRs) from multinomial logistic regression and adjusted prevalence ratios (aPRs) from modified Poisson models for BVDV and IBRV antibody coinfection status.

Outcome category	Model	aRRR	95% CI lower	95% CI upper	*p*	aPR	95% CI lower (aPR)	95% CI upper (aPR)	*p* (aPR)
BVDV−/IBRV−	Reference	—	—	—	—	—	—	—	—
BVDV+/IBRV−	Multinomial logit	0.865	0.708	1.059	0.16	1.057	0.922	1.213	0.43
BVDV−/IBRV+		0.500	0.318	0.785	<0.01	0.617	0.384	0.992	0.05
BVDV+/IBRV+		0.780	0.679	0.896	<0.001	0.956	0.924	0.996	0.01

*Note:* Reference: BVDV−/IBRV−; coefficients are shown with 95% CI and *p* values.

The strongest association was observed for BVDV−/IBRV+ (aRRR 0.500, 95% CI 0.318–0.785; *p*  < 0.01), followed by BVDV+/IBRV+ (aRRR 0.780, 95% CI 0.679–0.896; *p*  < 0.001) and BVDV+/IBRV− (aRRR 0.865, 95% CI 0.708–1.059; *p*  < 0.2). Confidence intervals did not cross 1.00, and the ranking of effects was stable across adjustments, indicating that discordant IBRV positivity contributed most to the departure from the double‐negative reference, with a secondary contribution from double positivity and a smaller effect for BVDV‐only positivity.

### 3.4. Antigen Positivity and Adjusted Associations BVDV and IBRV

Among 6651 clinical specimens tested, the overall antigen prevalence was 7.01% (95% CI: 6.40–7.65) for BVDV and 5.79% (95% CI: 5.24–6.38) for IBRV (Table 6), indicating substantially lower detection rates than corresponding seroprevalence.

**Table 6:** Prevalence of BVDV and IBRV antigens by province, year, season, and clinical status.

Distribution	Item	BVDV Ag % (95% CI)	IBRV Ag % (95% CI)
Overall	All	7.01 (6.40–7.65)	5.79 (5.24–6.38)
Province	Hebei	7.73 (6.45–9.17)	7.47 (6.22–8.90)
	Liaoning	5.62 (4.13–7.45)	4.74 (3.38–6.45)
	Inner Mongolia	6.01 (5.07–7.07)	4.24 (3.45–5.16)
	Sichuan	7.77 (6.35–9.40)	6.17 (4.90–7.65)
	Chongqing	8.63 (6.76–10.81)	7.36 (5.64–9.41)
Year	2022	6.20 (5.20–7.33)	4.75 (3.87–5.76)
	2023	7.28 (6.25–8.43)	5.38 (4.49–6.39)
	2024	7.45 (6.42–8.60)	7.11 (6.10–8.23)
Season	Spring	5.76 (4.51–7.22)	5.67 (4.44–7.13)
	Summer	5.54 (4.11–7.19)	5.28 (3.92–6.94)
	Autumn	7.81 (6.63–9.14)	5.96 (4.92–7.14)
	Winter	7.53 (6.56–8.59)	5.90 (5.04–6.85)
Clinic status	No	6.30 (5.42–7.26)	4.33 (3.60–5.15)
	Yes	7.52 (6.71–8.40)	6.85 (6.07–7.69)

*Note:* Values are % with 95% confidence intervals; *n* per stratum provided. Assays as described in methods.

BVDV antigen was highest in the southwest (e.g., Chongqing 8.63% and Sichuan 7.77%) compared to northern provinces (e.g., Liaoning 5.62% and Inner Mongolia 6.01%), with winter showing the greatest detection; IBRV antigen displayed a similar seasonal pattern and clustered among symptomatic cattle. Stratified estimates are provided in Table 6, Figure 5.

Figure 5Prevalence of BVDV and IBRV antigens by province, year, season, and clinical status. (A) Spatial distribution of BVDV and IBRV antigen positivity in cattle across Chinese provinces. (B) Comparison of BVDV and IBRV antigen positivity by epidemiological factors. (C) Forest plot of BVDV antigen positivity by epidemiological factors. (D) Forest plot of IBRV antigen positivity by epidemiological factors. (A–D) BVDV and IBRV; bars/points depict proportions with 95% CI. Overall antigen positivity: BVDV 7.01% (95% CI 6.40–7.65); IBRV 5.79% (95% CI 5.24–6.38).

**Figure:**
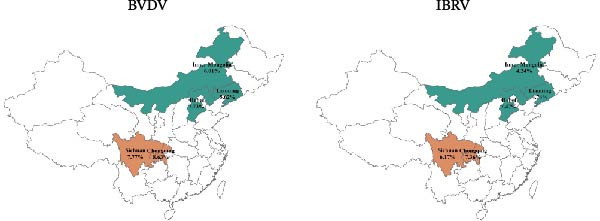
(A)

**Figure:**
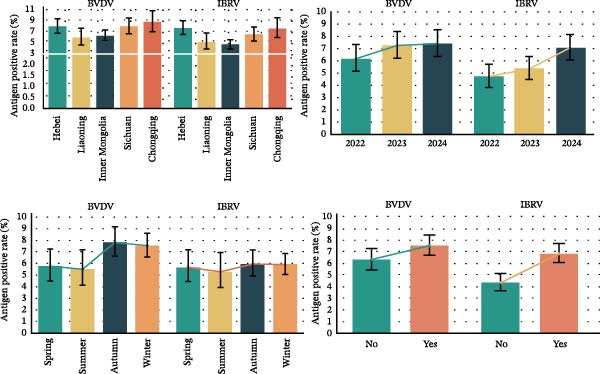
(B)

**Figure:**
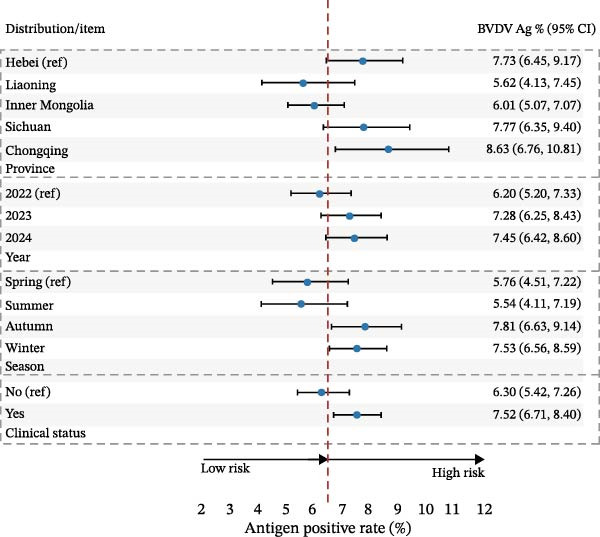
(C)

**Figure:**
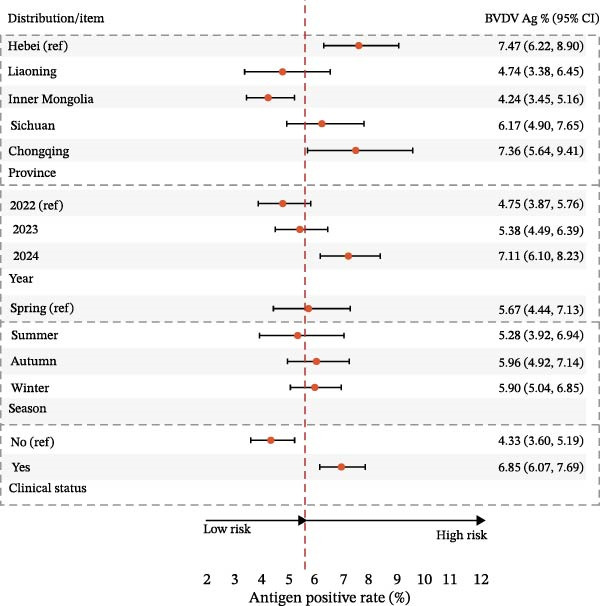
(D)

In multivariable logistic models adjusting for province, year, season, and clinical status, BVDV antigen remained higher in Sichuan/Chongqing than Hebei, with elevated levels also observed during winter relative to spring. In contrast, the association between BVDV antigen detection and clinical signs was weak and inconsistent across stratified analyses. For IBRV antigen, there was a temporal increase in 2024 vs. 2022 (aOR 1.534, 95% CI 1.163–2.023, *p*  < 0.05) and a strong association with clinical signs (aOR 1.958, 95% CI 1.536–2.495, *p*  < 0.001). Geographic and seasonal terms for IBRV were modest after adjustment. Full coefficients are reported in Table 7, with corresponding forest plots in Figure 6.

Figure 6Multivariable logistic regression analyses of potential risk factors associated with BVDV and IBRV Ag positivity. (A) Forest plot of adjusted odds ratios (aORs) for BVDV antigen positivity by epidemiological factors. (B) Forest plot of adjusted odds ratios (aORs) for IBRV antigen positivity by epidemiological factors. Forest plots display aORs with 95% CI; dashed vertical line at 1.00. Examples (IBRV Ag): 2024 vs. 2022 aOR 1.534 (1.163–2.023); clinical signs (Yes) aOR 1.958 (1.536–2.495).

**Figure:**
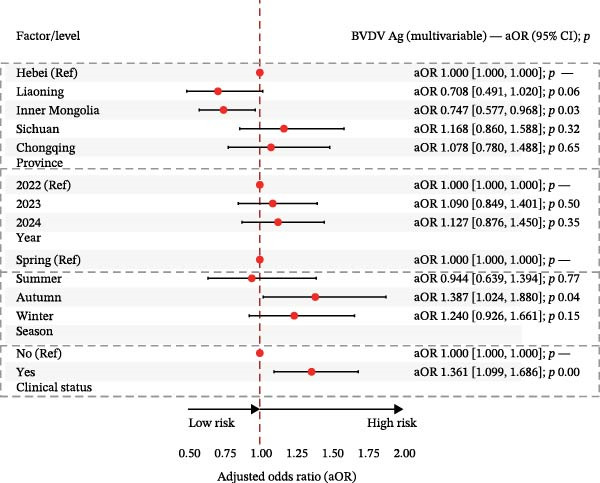
(A)

**Figure:**
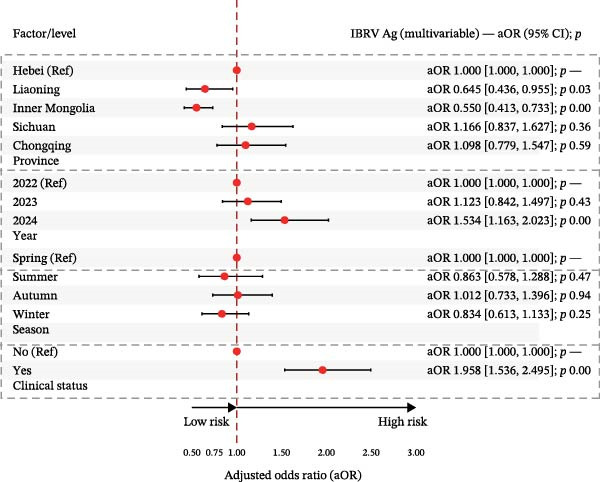
(B)

**Table 7:** Univariable and multivariable logistic regression for BVDV and IBRV antigen positivity.

Factor	Level	OR (95% CI)	*p*	aOR (95% CI)	aP	OR (95% CI)	*p*	aOR (95% CI)	aP
		BVDV				IBRV			
Province	Hebei (ref)	1	—	1	—	1	—	1	—
	Liaoning	0.71 (0.499–1.012)	0.06	0.708 (0.491–1.020)	0.06	0.617 (0.423–0.899)	0.01	0.645 (0.436–0.955)	0.03
	Inner Mongolia	0.763 (0.592–0.985)	0.04	0.747 (0.577–0.968)	0.03	0.549 (0.415–0.725)	<0.001	0.55 (0.413–0.733)	<0.001
	Sichuan	1.006 (0.761–1.329)	0.7	1.168 (0.860–1.588)	0.32	0.814 (0.604–1.097)	0.18	1.166 (0.837–1.627)	0.36
	Chongqing	1.127 (0.826–1.538)	0.45	1.078 (0.780–1.488)	0.65	0.984 (0.709–1.365)	0.92	1.098 (0.779–1.547)	0.594
Year	2022 (ref)	1	—	1	—	1	—	1	—
	2023	1.188 (0.935–1.508)	0.16	1.09 (0.849–1.401)	0.50	1.142 (0.869–1.500)	0.34	1.123 (0.842–1.497)	0.43
	2024	1.218 (0.961–1.543)	0.10	1.127 (0.876–1.450)	0.35	1.54 (1.187–1.986)	0.001	1.53 (1.163–2.023)	0.002
Season	Spring (ref)	1	—	1	—	1	—	1	—
	Summer	0.953 (0.656–1.384)	0.80	0.944 (0.639–1.394)	0.77	0.927 (0.634–1.354)	0.70	0.863 (0.578–1.288)	0.47
	Autumn	1.388 (1.033–1.865)	0.03	1.387 (1.024–1.880)	0.04	1.053 (0.772–1.436)	0.75	1.012 (0.733–1.396)	0.94
	Winter	1.333 (1.007–1.765)	0.05	1.24 (0.926–1.661)	0.15	1.042 (0.779–1.394)	0.78	0.834 (0.613–1.133)	0.25
Clinical status	No (ref)	1	—	1	—	1	—	1	—
	Yes	1.21 (0.997–1.469)	0.05	1.361 (1.099–1.686)	0.005	1.624 (1.302–2.026)	<0.001	1.958 (1.536–2.495)	<0.001

*Note:* aOR with 95% CI and *p* values; models adjust for province, year, season, and clinical status.

### 3.5. Antigen Coinfection (Multinomial Model)

Using BVDV−/IBRV− as the reference and adjusting for province, year, season. and clinical status, multinomial models showed higher relative risk for all nonreference categories (Table 8, Figure 7).

Figure 7Adjusted relative risk ratios (aRRRs) and adjusted prevalence ratios (aPRs) for antigen coinfection. (A) Adjusted relative risk ratios (aRRRs) for BVDV–IBRV coinfection. (B) Adjusted prevalence ratios (aPRs) for BVDV–IBRV coinfection. Outcome categories: BVDV−/IBRV− (reference), BVDV+/IBRV−, BVDV−/IBRV+, and BVDV+/IBRV+. Points = aRRR (or aPR where shown); bars = 95% CI; dashed vertical line at 1.00. Largest effect observed for BVDV−/IBRV+.

**Figure:**
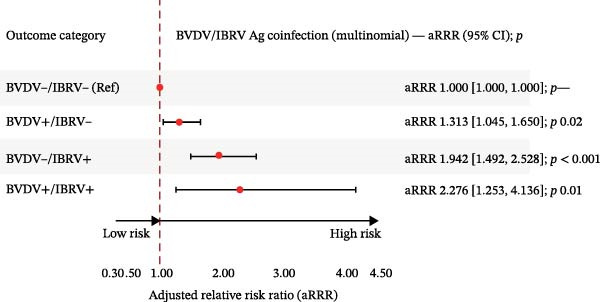
(A)

**Figure:**
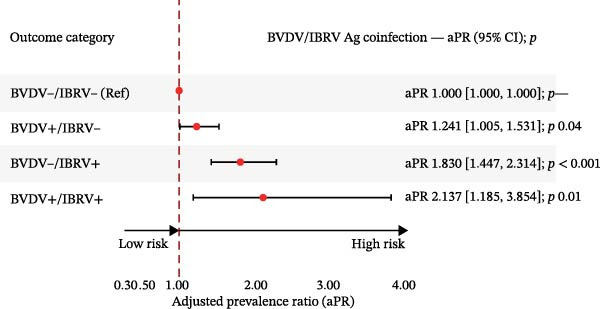
(B)

**Table 8:** Adjusted relative risk ratios (aRRRs) from multinomial logistic regression and adjusted prevalence ratios (aPRs) from modified Poisson models for BVDV and IBRV antigen coinfection status.

Outcome category	Model	aRRR	95% CI lower	95% CI upper	*p*	aPR	95% CI lower (aPR)	95% CI upper (aPR)	*p* (aPR)
BVDV−/IBRV−	Reference	—	—	—	—	—	—	—	—
BVDV+/IBRV−	Multinomial logit	1.313	1.045	1.650	0.02	1.241	1.005	1.531	0.04
BVDV−/IBRV+		1.942	1.492	2.528	<0.001	1.83	1.447	2.314	<0.001
BVDV+/IBRV+		2.276	1.253	4.136	0.01	2.137	1.185	3.854	0.01

*Note:* Reference: BVDV−/IBRV−; coefficients are shown with 95% CI and *p* values.

The IBRV‐only exhibited the strongest association with the reference category, followed by double‐positive (BVDV+/IBRV+), with BVDV‐only elevated to a lesser extent. Effects were most apparent in the southwest and during winter, and clinical signs were consistently linked to codetection—particularly for strata involving IBRV. A modest temporal increase was evident in 2024 compared to 2022. Overall, antigen coinfection patterns mirror the serologic findings, with IBRV contributing more to discordant positivity and double positivity in this cohort.

## 4. Discussion

Although the epidemiology of BRDC is highly complex in transport‐associated, with tremendous variability in disease expression resulting from interactions among pathogen, host, and management factors [23]. This multiprovincial survey demonstrates that BVDV and IBRV remain entrenched in China’s cross‐regional finishing system. Seropositivity was high and geographically structured, with consistently greater burdens in southwestern receiving hubs and a winter peak (Table 3; Figure 2). These patterns persisted in adjusted models (Table 4; Figure 3) and were echoed—though at much lower prevalence—by antigen detection (Tables 6 and 7; Figures 5 and 6). Collectively, the data point to movement‐linked transmission with seasonal amplification.

### 4.1. Geography, Time, and Season

Southwestern provinces (Sichuan/Chongqing) showed higher odds of BVDV seropositivity versus Hebei after adjustment, and IBRV rose in 2024 relative to 2022 (Table 4). The long‐distance movement of cattle introduces immunologically naïve and potentially infected animals into new populations, increasing both viral shedding (reflected in antigen detection) and seroconversion rates. The observed geographic concentration at finishing hubs is consistent with this mechanism, driven by large‐volume commingling and extended residence time posttransport [1]. The winter elevation fits known constraints on housing and ventilation during cold weather. While cross‐sectional data do not permit causal attribution, the internal coherence across antibodies and antigens (Figures 2, 3, 5, and 6) strengthens the inference that both location in the chain and calendar period matter for risk. Human respiratory syncytial virus (RSV) exhibits distinct geographical and seasonal patterns [24], indicating these factors should be considered key determinants in viral transmission mechanisms.

### 4.2. Clinical Status and Diagnostic Pairing

Animals with clinical signs had lower odds of seropositivity yet higher odds of IBRV antigen positivity after adjustment (Tables 4 and 7). This seemingly paradoxical finding has biological plausibility. First, as primary pathogens, BVDV and IBRV are known to cause immunosuppression or establish latent infections. During the initial stages of acute infection or stress‐induced reactivation, the host’s humoral immune response may not be fully established or may be waning [25]. Second, antigen/PCR detection directly indicates active infection, whereas serological testing reflects prior immune experience; the two markers inherently represent different stages of the infection process [26]. Consequently, in practice, implementing a paired diagnostic strategy at intake—using antigen/PCR for rapid identification of actively infected individuals, while employing serology to assess the herd’s overall immune status and guide vaccination protocols—has proven to be an effective management approach [27]. For practice, intake screening is best served by paired diagnostics—targeted antigen/PCR for case finding, plus serology to gauge immunity gaps and guide boosters—especially before winter and in high‐throughput yards.

### 4.3. Coinfection

Multinomial models referenced to the double‐negative group indicated a graded pattern for antibodies: the strongest association for IBRV‐only, followed by double‐positive, with BVDV‐only elevated to a lesser extent (Table 5; Figure 4). Antigen coinfection showed the same directionality, with effects tighter among symptomatic cattle and a modest temporal increase in 2024 (Table 8; Figure 7). These results suggest that IBRV contributes disproportionately to discordant and concurrent positivity within this network, a finding aligned with its role in BRDC clinical presentation. IBRV is a primary respiratory pathogen, renowned for its high replicative capacity in the upper respiratory tract and strong cytopathic effects, enabling it to directly induce significant clinical signs. This explains its strongest direct association with the disease phenotype [28]. In contrast, the pathogenesis of BVDV centers on its broad immunosuppressive effects; while it is crucial to the overall occurrence of BRDC, singular infection may not directly cause severe clinical symptoms, thus manifesting as a weaker direct effect in statistical models [29]. In coinfection scenarios, the immunosuppressive environment created by BVDV likely amplifies the pathogenicity of IBRV. This may account for the intermediate risk level observed in the double‐positive group and underscores the complex interactions between these pathogens [30]. Consequently, our data confirm that targeted control strategies should prioritize the surveillance and management of IBRV transmission.

### 4.4. Implications for Control

Three priorities emerge. First, at origin, verify BVDV and IBRV vaccination status, remove BVDV PI animals, and limit supplier mixing. Second, at arrival, apply a 48–72 h period cohorting by source with paired diagnostics, and escalate monitoring during winter. Third, by region/season, allocate testing and vaccine logistics preferentially to southwestern hubs and the prewinter window. These steps are intended as pragmatic, evidence‐informed recommendations that align with existing livestock movement logistics.

### 4.5. Strengths and Limitations

Key strengths of this study are the large multiprovincial sample, integrated antibody and antigen testing, and formal modeling of coinfection patterns. Limitations include that the cross‐sectional nature of this study limits causal inference; therefore, the observed associations should be interpreted as statistical rather than definitive causal relationships. Second, the absence of individual animal vaccination and treatment histories may have introduced misclassification in serological and virological prevalence estimates, affecting the precision of our interpretations. Third, although several key covariates were adjusted for in the analyses, potential confounders such as detailed transport conditions and pen‐level management practices were not captured, leaving the possibility of residual confounding. Finally, the inherent clustering of samples—by consignment, market, or farm—was not explicitly accounted for through random effects in our primary models, which may have influenced the precision of parameter estimates. Future work should use longitudinal cohorts from preshipment through early finishing, incorporate mixed‐effects or hierarchical models to capture clustering, and integrate genomic or movement data to trace introductions and onward spread.

## 5. Conclusion

Within China’s growing cross‐regional finishing model, BVDV and IBRV remain movement‐sensitive hazards with southwestern concentration, winter amplification, and recent temporal increase. Risk‐stratified vaccination, PI testing at origin, strengthened arrival biosecurity, and calendar‐aware surveillance are justified by the present evidence.

## Author Contributions

Conceptualization, writing – original draft: Li Ren, Cheng Shen, Changxiao Tian, Xijun Yan, and Zhicai Zuo. Methodology, data curation, software: Li Ren, Cheng Shen, Yanxia Hao, and Xijun Yan. Investigation, formal analysis: Li Ren and Yanxia Hao. Writing – review and editing: all authors. Funding acquisition, project administration: Zhicai Zuo and Xijun Yan.

## Funding

This work was supported by the China Agriculture Research System of MOF and MARA (Beef Cattle/Yak, Grant CARS‐37).

## Ethics Statement

All experimental procedures in this study were conducted in accordance with the ARRIVE guidelines and Chinese laws.

## Conflicts of Interest

The authors declare no conflicts of interest.

## Data Availability

All data and material can be accessed from the corresponding author upon request from the corresponding author.
